# CEACAM1 promotes CD8^+^ T cell responses and improves control of a chronic viral infection

**DOI:** 10.1038/s41467-018-04832-2

**Published:** 2018-07-02

**Authors:** Vishal Khairnar, Vikas Duhan, Ashwini M. Patil, Fan Zhou, Hilal Bhat, Christine Thoens, Piyush Sharma, Tom Adomati, Sarah-Kim Friendrich, Judith Bezgovsek, Janine D. Dreesen, Gunther Wennemuth, Astrid M. Westendorf, Gennadiy Zelinskyy, Ulf Dittmer, Cornelia Hardt, Jörg Timm, Joachim R. Göthert, Philipp A. Lang, Bernhard B. Singer, Karl S. Lang

**Affiliations:** 1 Institute of Immunology, University Hospital Essen, Hufelandstrasse 55, 45147 Essen, Germany; 2 Department of Hematology, West German Cancer Center (WTZ), University Hospital Essen, Hufelandstrasse 55, 45147 Essen, Germany; 3 Institute of Virology, Medical Faculty, Heinrich-Heine-University, Universitätsstrasse 1, 40225 Düsseldorf, Germany; 4 Institute of Anatomy, Medical Faculty, University Hospital Essen, Hufelandstrasse 55, 45147 Essen, Germany; 5 Institute of Medical Microbiology, Faculty of Medicine, University Hospital Essen, Hufelandstrasse 55, 45122 Essen, Germany; 6 Institute for Virology, University Hospital Essen, Hufelandstrasse 55, 45147 Essen, Germany; 7 Clinic of Gastroenterology, Hepatology and Infectious Disease, Heinrich-Heine-University, Moorenstrasse 5, 40225 Düsseldorf, Germany; 8 Department of Molecular Medicine II, Medical Faculty, Heinrich-Heine-University, Universitätsstrasse 1, 40225 Düsseldorf, Germany

## Abstract

Dysfunction of CD8^+^ T cells can lead to the development of chronic viral infection. Identifying mechanisms responsible for such T cell dysfunction is therefore of great importance to understand how to prevent persistent viral infection. Here we show using lymphocytic choriomeningitis virus (LCMV) infection that carcinoembryonic antigen-related cell adhesion molecule 1 (CEACAM1) is fundamental for recruiting lymphocyte-specific protein kinase (Lck) into the T cell receptor complex to form an efficient immunological synapse. CEACAM1 is essential for activation of CD8^+^ T cells, and the absence of CEACAM1 on virus-specific CD8^+^ T cells limits the antiviral CD8^+^ T cell response. Treatment with anti-CEACAM1 antibody stabilizes Lck in the immunological synapse, prevents CD8^+^ T cell exhaustion, and improves control of virus infection in vivo. Treatment of human virus-specific CD8^+^ T cells with anti-CEACAM1 antibody similarly enhances their proliferation. We conclude that CEACAM1 is an important regulator of virus-specific CD8^+^ T cell functions in mice and humans and represents a promising therapeutic target for modulating CD8^+^ T cells.

## Abstract (web summary)

Chronic viral infections are frequently associated with the dysfunction of CD8^+^ T cells which includes loss of function and results in CD8^+^ T cell exhaustion. Here the authors show a role of CEACAM1 in promoting responsive CD8^+^ T cells in the context of a chronic lymphocytic choriomeningitis virus (LCMV) infection model.

## Introduction

Chronic viral infections are an important worldwide health concern. Globally, approximately 240 million people are chronically infected with hepatitis B virus (HBV)^1^, more than 170 million are infected with hepatitis C virus (HCV)^2,3^, and 75 million are infected with human immunodeficiency virus (HIV)^4^. These viruses can lead to persistent infection and dysfunction of adaptive immunity^2,5^. Although HCV infection can be cured with antiviral therapy, treatment for HIV and HBV does not result in complete clearance of the virus. Therefore, patients must take antiviral drugs every day to prevent severe clinical problems such as AIDS or end-stage liver failure^5,6^. Thus, identifying new strategies that result in complete control of virus is one main goal of basic research.

One important reason for immune dysfunction is the abrogation of the function of virus-specific CD8^+^ T cells. Typically, in T cell dysfunction CD8^+^ T cells lose their ability to proliferate and to produce cytokines, such as tumor necrosis factor-α (TNF) and interferon-γ (IFN-γ). Effector and memory functions are also substantially lost. Next, CD8^+^ T cells undergo apoptosis and are eliminated by phagocytic cells in a hierarchical manner, called T cell exhaustion^7,8^. Stimulation through the T cell receptor (TCR) triggers phosphorylation signals that not only activate multiple effector pathways but also ensure that T cells respond to an appropriate signal. CD28 family members are mostly co-stimulatory and provide an activation signal via TCR^9^. Besides positive activation, negative regulators such as programmed cell death 1 (PD-1) and T cell immunoglobulin and mucin domain containing 3 (TIM3) signal via TCR and by various cell surface receptors^9,10^. PD-1 is an important inhibitory receptor that induces the exhaustion of CD8^+^ T cells. Engagement of PD-1 by its ligands PD-L1 or PD-L2 produces signals that inhibit T cell proliferation and function^11,12^. Blockade of PD-1 restores the function of exhausted CD8^+^ T cells and can therefore be used to treat diseases that profit from restored CD8^+^ T cell function^13–15^. Because of the clinical success of PD-1 blockade in humans, current research focuses on the discovery of other checkpoint regulators of CD8^+^ T cells.

Carcinoembryonic antigen-related cell adhesion molecule 1 (CEACAM1, CD66a, or biliary glycoprotein) is a member of the carcinoembryonic antigen (CEA) family. It is engaged in cell–cell communication that affects various signal transduction processes associated with cell activation, proliferation, differentiation, and apoptosis^16–18^. CEACAM1 usually acts via intercellular adhesion through N-domain-mediated homophilic (CEACAM1-CEACAM1) or heterophilic (CEACAM1-CEACAM5, 6, or 8) interactions^19–22^. Despite its numerous functions and expression on a broad range of cell types, such as microvascular endothelia, epithelial cells, immune cells including T and B cells, macrophages, dendritic cells, and tumor cells^16,18^, *Ceacam1*^*–/–*^ mice develop normally^23^.

The long isoform of CEACAM1 (CEACAM1-L) contains two immunoreceptor tyrosine-based inhibitory motifs (ITIMs)^24^. Therefore, CEACAM1 was believed to be an inhibitory receptor^25^ that suppresses the activation of CD4^+^ T cells^26,27^. CEACAM1-L and CEACAM1-S variants can undergo dimerization depending on the intracellular calcium (Ca^2+^) concentration^16,28^. Interestingly, long and short cytoplasmic variants of CEACAM1 provide both inhibitory and activatory signals^16,29–31^. CEACAM1-4S alone in the absence of CEACAM1-4L has also been shown to promote the induction of regulatory T cells and follicular helper T cells (Tfh cells) in the intestine^27^. In contrast, it has been shown that CEACAM1 interacts with TIM3 and thereby suppresses CD4^+^ T cell activation^32^. Besides its direct involvement in phosphorylation and calcium signaling, CEACAM1 interacts with filamin A and therefore modulates the topology of the signaling complex^33^. In CD8^+^ T cells, filamin A interacts with CD28 and lymphocyte-specific protein kinase (Lck) and is therefore an important molecule that modulates membrane rearrangement and CD8^+^ T cell signaling^34^. Whether CEACAM1 interacts with filamin A in T cells and thereby contributes to CD8^+^ T cell function remains unknown.

The study described here, using the LCMV mouse model, demonstrates that CEACAM1 is expressed on virus-specific CD8^+^ T cells. The absence of CEACAM1 on CD8^+^ T cells limits CD8^+^ T cell expansion and function and results in viral persistence. The absence of CEACAM1 leads to decreased TCR activation and limits the phosphorylation of ζ-chain-associated protein kinase 70 (Zap-70), phospholipase Cγ1 (PLCγ-1), and extracellular signal-regulated kinase (Erk). Mechanistically, CEACAM1 is fundamental for recruiting Lck to the immunological synapse. Notably, treatment with anti-CEACAM1 antibody stabilizes Lck in the immunological synapse and restores the function of exhausted CD8^+^ T cells.

## Results

### CEACAM1 is important in clearing LCMV infection

**Fig. 1:**
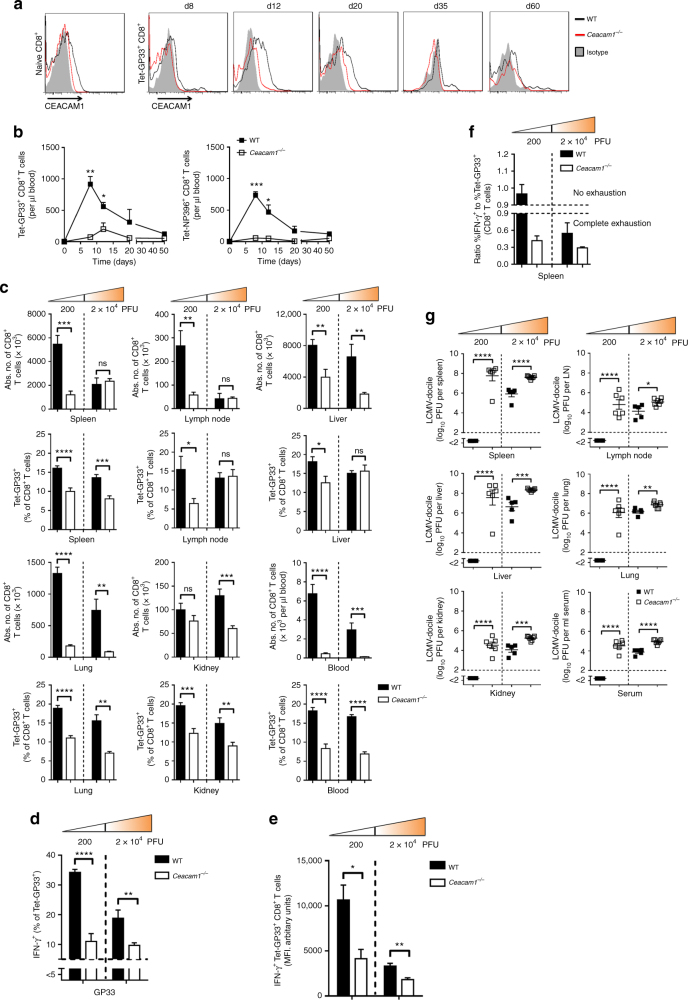
CEACAM1 is expressed on CD8^+^ T cells and is essential for resolving LCMV infection. **a** CEACAM1 expression on naïve CD8^+^ T cells (left panel) and Tet-GP33^+^ CD8^+^ T cells (right panel) from wild-type (WT; black line) or *Ceacam1*^*–/–*^ (red line) mice infected with 2 × 10^4^ plaque-forming units (PFU) of LCMV-Docile at indicated time points. Isotype control antibody staining of T cells from WT mice is shown as a gray area (*n* = 4–6 per group). **b** Absolute number of Tet-GP33^+^ and NP396^+^ CD8^+^ T cells in blood from WT or *Ceacam1*^*–/–*^ mice after intravenous infection with 200 PFU of LCMV-Docile at indicated time points (*n* = 4–6 per group). **c** Absolute number of CD8^+^ T cells and percentages of Tet-GP33^+^ CD8^+^ T cells in spleen, lymph node, liver, lung, kidney, and blood (*n* = 6–8 per group) from WT or *Ceacam1*^*–/–*^ mice on day 8 after infection with 200 or 2 × 10^4^ PFU of LCMV-Docile. **d**, **e** Intracellular cytokine interferon-γ (IFN-γ) secretion in Tet-GP33^+^ CD8^+^ T cells (**d**) and median fluorescence intensity (MFI) levels in IFN-γ^+^ Tet-GP33^+^ CD8^+^ T cells (**e**) from splenocytes of WT and *Ceacam1*^*–/–*^ mice on day 8 after infection with 200 or 2 × 10^4^ PFU of LCMV-Docile. Horizontal dotted lines designate the detection limit without restimulation (*n* = 6–8 per group). **f** Ratio of the percentage of IFN-γ^+^ cells to the percentage of Tet-GP33^+^ CD8^+^ T cells from WT and *Ceacam1*^*–/–*^ mice on day 8 after infection with 200 (*n* = 12) or 2 × 10^4^ (*n* = 7) PFU of LCMV-Docile. **g** Viral titers from WT and *Ceacam1*^*–/–*^ mice in indicated organs and serum 8 days after intravenous infection with 200 or 2 × 10^4^ PFU of LVMC-Docile (*n* = 6–8 per group). *P *<* 0.05; **P < 0.01; ***P < 0.001; and ****P < 0.0001 (Student’s *t* test). ns = not significant. Data are representative of two (**a**, **c**, **d**, **e**, **f**, **g**) or three (**b**) experiments (mean ± SEM; **b**, **c**, **d**, **e**, **f**, **g**)

In mice, LCMV strain WE causes an acute infection that can be cleared within 1 to 2 weeks because of efficient induction of virus-specific cytotoxic T lymphocytes (CTLs), whereas a higher dose of LCMV strain Docile persists (Supplementary Fig. 1a), causing chronic infection and persistent inflammation^8,35^. To obtain insights into the potential role of CEACAM1 in CD8^+^ T cell responses during chronic viral infection, we challenged C57BL/6 (wild-type, WT) and *Ceacam1*^*–/–*^ mice with a viral dose leading to a persistent infection with LCMV-Docile and measured the expression of CEACAM1 on virus-specific CD8^+^ T cells. Naïve peripheral CD8^+^ T cells exhibited little expression of CEACAM1 (Fig. 1a, left panel). After infection with LCMV-Docile, LCMV-GP33-specific CD8^+^ T cells also expressed higher levels of CEACAM1. The expression level was at its highest on day 12 after infection (Fig. 1a, right panel). However, 35 days after infection only a fraction of these virus-specific CD8^+^ T cells stained positive for CEACAM1 (Fig. 1a, right panel).

To elucidate the function of CEACAM1 on CD8^+^ T cells during viral infection, we challenged WT and *Ceacam1*^*–/–*^ mice with 200 plaque-forming units (PFU) of LCMV-Docile. The absence of CEACAM1 led to reduced expansion of virus-specific GP33^+^ and NP396^+^ CD8^+^ T cells in blood, as measured by major histocompatibility (MHC) I tetramers (Fig. 1b). Similarly, the number of CD8^+^ T cells and the percentage of virus-specific Tet-GP33^+^ CD8^+^ T cells were reduced in peripheral organs and blood of *Ceacam1*^*–/–*^ mice 8 days after infection with 200 PFU of LCMV-Docile (Fig. 1c). A similar trend was observed upon infection with 2 × 10^4^ PFU of LCMV-Docile. A higher antigen load led to a gradual decrease in the total number of CD8^+^ T cells and in the percentage of virus-specific Tet-GP33^+^ CD8^+^ T cells in WT mice infected with 2 × 10^4^ PFU of LCMV-Docile compared to the mice infected with 200 PFU of LCMV-Docile (Fig. 1c and Supplementary Fig. 1b).

The absence of CEACAM1 also caused loss of CD8^+^ T cell function, because the production of IFN-γ by virus-specific Tet-GP33^+^ CD8^+^ T cells (Fig. 1d and Supplementary Fig. 1c) and by the total of CD8^+^ T cells (Supplementary Fig. 1d and 1e) was blunted in *Ceacam1*^*–/–*^ mice after in vitro restimulation of splenocytes with virus-specific GP33 peptide. This finding clearly suggests that the remaining virus-specific Tet-GP33^+^ CD8^+^ T cells in *Ceacam1*^*–/–*^ mice produce reduced amounts of IFN-γ (Fig. 1d).

In line with this finding, the median fluorescence intensity levels of IFN-γ from virus-specific Tet-GP33^+^ CD8^+^ T cells (Fig. 1e) and from total CD8^+^ T cells (Supplementary Fig. 1f) were also reduced in *Ceacam1*^*–/–*^ mice infected with 200 or 2 × 10^4^ PFU of LCMV-Docile after in vitro restimulation of splenocytes with virus-specific GP33 peptide. In WT mice an increase in antigen load decreased both the percentage of virus-specific Tet-GP33^+^ CD8^+^ T cells and the production of IFN-γ after restimulation, leading to CD8^+^ T cell exhaustion (Fig. 1c, d and Supplementary Fig. 1b–e). However, in *Ceacam1*^*–/–*^ mice these CD8^+^ T cell functions were impaired by infection with either amount of LCMV-Docile PFUs, because the ratio of the percentage of IFN-γ^+^ T cells to the percentage of Tet-GP33^+^ CD8^+^ T cells was reduced and was almost equally independent of antigen load, a finding suggesting that the lack of CEACAM1 renders CD8^+^ T cells sensitive to exhaustion (Fig. 1f).

Surface expression of CD107a and CD107b is associated with the release of lytic granule proteins such as granzyme B and perforin and is a surrogate marker of degranulation-dependent cytotoxicity^36^. However, the expression of CD107a on total CD8^+^ T cells, on CD8^+^ T cells with the effector phenotype (CD43^+^ CD62L^–^), and on virus-specific Tet-GP33^+^ CD8^+^ T cells was higher in *Ceacam1*^*–/–*^ mice than in WT mice (Supplementary Fig. 2a). Also, the production of cytolytic granule granzyme B was slightly higher in virus-specific Tet-GP33^+^ CD8^+^ T cells in *Ceacam1*^*–/–*^ mice than in WT mice (Supplementary Fig. 2b). During LCMV infection, the ability to eliminate infected cells depends primarily on CD8^+^ T cells. Reduced CD8^+^ T cell expansion and function in *Ceacam1*^*–/–*^ mice was associated with impaired control of virus, because viral titers were significantly higher in peripheral organs and serum of *Ceacam1*^*–/–*^ mice 8 days after infection, whereas WT mice exhibited undetectable or significantly lower levels of infectious particles (Fig. 1g).

Next, we determined whether the absence of CEACAM1 restricts the expansion of CD8^+^ T cells during other acute viral infections. To do so, we infected WT and *Ceacam1*^*–/–*^ mice with 200 PFU of LCMV strain WE. The total number of CD8^+^ T cells, virus-specific Tet-GP33^+^ CD8^+^ T cell expansion (Supplementary Fig. 3a), and intracellular cytokine (IFN-γ and TNF) production (Supplementary Fig. 3b) was limited in *Ceacam1*^*–/–*^ mice 8 days after infection. Nevertheless, *Ceacam1*^*–/–*^ mice were able to control infection with 200 PFU of LCMV-WE at day 8 after infection (Supplementary Fig. 3c). Altogether, we conclude that CEACAM1 expression is essential for the induction, expansion, and function of virus-specific CD8^+^ T cells and the control of chronic viral infection.

### CEACAM1 is necessary for maintaining splenic architecture

**Fig. 2:**
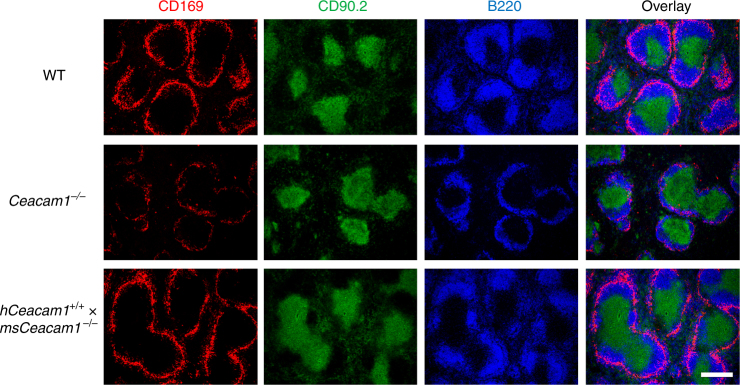
Lack of CEACAM1 influences splenic architecture. Representative immunofluorescence of sections from spleens of naive wild-type (WT), *Ceacam1*^*–/–*^, and *hCeacam1*^+/+^ × *msCeacam1*^*–/–*^ mice after staining for marginal zone macrophages (CD169, red), T cells (CD90.2, green), and B cells (B220, blue) (*n* = 4–5 per group). Scale bar, 300 µm. One slide representative of four or five slides is shown

Control of LCMV depends on the interplay of several innate and adaptive immune cells. Of special importance are the marginal zone macrophages and marginal zone metallophils, which capture LCMV within minutes after infection^37^. Thereby marginal zone macrophages prevent viral spread to peripheral tissues and contribute to activation of the innate and adaptive immune system against LCMV^37^^,^^38^. Compared to WT mice, *Ceacam1*^*–/–*^ mice showed an aberrant splenic architecture with remarkable reduction in the number of total B cells and CD169^+^ marginal zone macrophages (Fig. 2). Lack of CD169^+^ macrophages as well as the reduced number of B cells may contribute to insufficient virus-specific CD8^+^ T cell responses in *Ceacam1*^*–/–*^ mice. Therefore, we hypothesized that CEACAM1 may also play a cell-intrinsic role in virus-specific CD8^+^ T cells.

### CD8^+^ T cell-intrinsic CEACAM1 is necessary for viral control

**Fig. 3:**
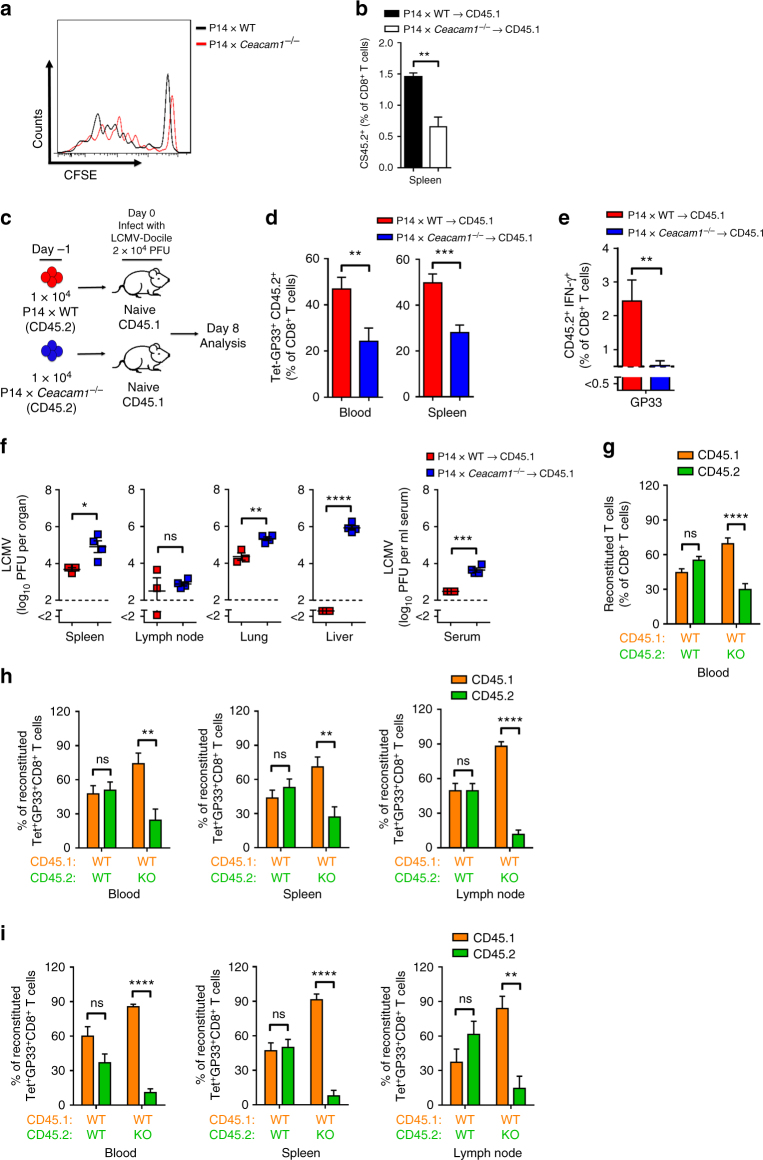
The action of CEACAM1 is intrinsic to T cells. Representative flow cytometry histogram of proliferating CD8^+^ T cells (**a**) and bar diagram showing percentage of proliferated CD8^+^ T cells (**b**) from P14 × wild-type (WT) (black line) or P14 × *Ceacam1*^*–/–*^ (red line) mice that were adoptively transferred into WT (CD45.1) mice (1 × 10^7^ cells per mouse) on day −1. Mice were infected with 200 PFU of LCMV-Docile on day 0, and splenic tissue samples were examined 4 days after infection. **c** Experimental setup. **d**–**f** We transferred 1 × 10^4^ P14 × WT or P14 × *Ceacam1*^*–/–*^ (CD45.2) splenocytes into naïve CD45.1 WT mice on day –1 and then infected them with 2 × 10^4^ PFU of LCMV-Docile on day 0, analyzed on day 8 after infection. **d** Percentage of Tet-GP33^+^ CD45.2^+^ CD8^+^ T cells in blood (*n* = 6–7) and in spleen (*n* = 10). **e** Intracellular cytokine IFN-γ secretion by splenocytes from P14 × WT or P14 × *Ceacam1*^*–/–*^ mice. Horizontal dotted lines designate the detection limit without restimulation (*n* = 6–7 per group). **f** Viral titers in indicated organs from CD45.1 WT mice (*n* = 3–4 per group). **g–i** Statistical analysis of CD8^+^ T cell population or Tet-GP33^+^ CD8^+^ T cells from murine bone marrow chimeras reconstituted with 1:1 composition of bone marrow from CD45.1 WT:CD45.2 WT mice in one group and bone marrow from CD45.1 WT:*Ceacam1*^*–/–*^ (CD45.2) mice in another group after 45 days of reconstitution before infection, as measured by flow cytometry in blood (**g**, *n* = 8 per group), and in blood, spleen, and lymph nodes 8 days after infection with 200 PFU (**h**) or 2 × 10^4^ PFU (**i**) of LCMV-Docile (*n* = 5 per group). *P *<* 0.05; **P *<* 0.01; ***P *<* 0.001; ****P *<* 0.0001 (Student’s *t* test). ns = not significant. Data are representative of two (**b**, **g–i**), or three (**d**, **e**) experiments or one of two independent (**a**, **f**) experiments (mean ± SEM; **b**, **d**–**i**)

To determine whether the reduced expansion of CD8^+^ T cells in *Ceacam1*^*–/–*^ mice is associated with reduced proliferation or survival of activated T cells, we crossed *Ceacam1*^*–/–*^ and WT mice with P14 mice, which carry an LCMV-GP33-specific TCR as a transgene. We adoptively transferred carboxy-fluorescein succinimidyl ester (CFSE)-labeled P14 × WT or P14 × *Ceacam1*^*–/–*^ splenocytes into WT host animals (CD45.1) and then infected them with LCMV-Docile. Activation of transferred virus-specific P14 CD8^+^ T cells induced proliferation that was comparable between P14 × WT or P14 × *Ceacam1*^*–/–*^ CD8^+^ T cells (Fig. 3a). Therefore, we conclude that the induction of proliferation is independent of CEACAM1 expression. However, lack of CEACAM1 limited the survival of CD8^+^ T cells after activation, as demonstrated by the finding that the number of transferred P14 × *Ceacam1*^*–/–*^ CD8^+^ T cells were significantly lower than those of the P14 × WT CD8^+^ T cells (Fig. 3b). To further determine whether CEACAM1 acts directly on the expansion of CD8^+^ T cells after activation, we transferred equal (1 × 10^4^) number of P14 × WT or P14 × *Ceacam1*^*–/–*^ splenocytes into WT host animals (CD45.1) and then infected them with 2 × 10^4^ PFU of LCMV-Docile (Fig. 3c). We found that P14 × *Ceacam1*^*–/–*^ CD8^+^ T cells exhibited less expansion than did control P14 × WT CD8^+^ T cells (Fig. 3d). This finding may be due to the poor survival of CEACAM1-deficient CD8^+^ T cells after activation. Similarly, CEACAM1-deficient P14 CD8^+^ T cells exhibited limited IFN-γ production after in vitro restimulation with GP33 peptide (Fig. 3e), and this reduction in CD8^+^ T cell expansion and IFN-γ production was associated with enhanced viral titers in spleen, lung, liver, and serum at day 8 after infection (Fig. 3f).

To determine whether CEACAM1 deficiency affects the development of T cells in vivo, we examined thymic and splenic T cell subpopulations in naïve WT and *Ceacam1*^*–/–*^ mice (Supplementary Fig. 4). We found that the deficiency in CEACAM1 had no important impact on either absolute thymic cell numbers or the development of CD4 and CD8 double-positive thymocytes (Supplementary Fig. 4a). However, the number of mature CD4^+^ TCRβ^high^ and CD8^+^ TCRβ^high^ T cells were lower in *Ceacam1*^*–/–*^ mice than in WT mice. In line with this observation, we observed lower number of mature T cells in the spleen of *Ceacam1*^*–/–*^ mice (Supplementary Fig. 4b) than in the spleen of WT mice. Therefore, we conclude that CEACAM1 affects the generation of mature T cells, possibly by constraining the TCR-dependent thymic-positive selection process.

To determine whether the CEACAM1-mediated effect on T cells is independent of viral infection, we used mixed bone marrow chimeras, produced by irradiating CD45.2 WT mice and then reconstituting their immune system with bone marrow from CD45.1 WT mice and CD45.2 WT (mixed 1:1) mice or from CD45.1 WT mice and CD45.2 *Ceacam1*^*–/–*^ (mixed 1:1) mice. In line with the results found with naïve mice, we found that CEACAM1 exerted a slight influence on naïve CD8^+^ T cell numbers in the peripheral blood of bone marrow-reconstituted naïve mice (Fig. 3g). Additionally, 8 days after infection with 200 (Fig. 3h) or 2 × 10^4^ PFU (Fig. 3i) of LCMV-Docile, the virus-specific Tet-GP33^+^ CD8^+^ T cells in blood, spleen, and lymph nodes of the second group of mice were derived mainly from CD45.1 WT mice. We conclude that signals intrinsic to CEACAM1 T cells are essential for the activation, survival, or both of virus-specific CD8^+^ T cells. Taken together, these findings indicate that cell-intrinsic expression of CEACAM1 is essential for the survival and efficient expansion of CD8^+^ T cells after viral infection.

### CEACAM1 modulates the topology of the TCR signaling complex

Next, we aimed to examine the molecular mechanism by which CEACAM1 influences CD8^+^ T cell expansion. It has been reported that CD28 interacts with filamin A and the Src family kinase Lck at the immunological synapse^34,39^. Recruitment of CD28 to the immunological synapse during TCR activation promotes membrane rearrangements that lead to cytoskeletal changes for organized signaling^39–41^. Thus, either the absence of CD28 activation or a deficiency in Lck results in limited phosphorylation of Zap-70 and PLCγ-1^42-44^. Interestingly, the cytoplasmic domain of CEACAM1-L interacts with filamin A^33^; thus, we postulated that CEACAM1 may be involved in the recruitment of Lck into the TCR signaling complex.

**Fig. 4:**
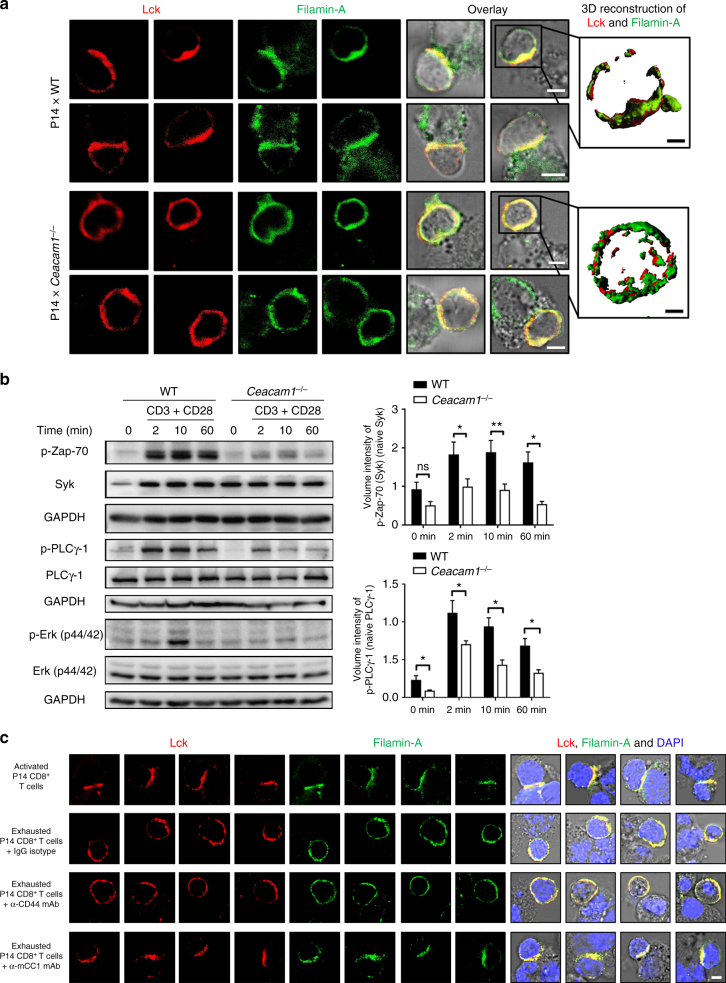
CEACAM1 is involved in the formation of the immunological synapse. **a** Immunofluorescence and 3D reconstruction of magnetic-activated cell sorting (MACS)-sorted CD8^+^ T cells from P14 × wild-type (WT) and P14 × *Ceacam1*^*–/–*^ mice; cells were cultured with GP33-loaded bone marrow-derived WT dendritic cells (DCs) for 30 minutes at 37 °C (*n* = 3) and then stained for Lck (red) and filamin A (green). Scale bars, 3 µm (main image) or 1 µm (insets). Images have been cropped for purposes of presentation. **b** Representative immunoblots and quantification of MACS-sorted WT and *Ceacam1*^*–/–*^ T cells after challenge with anti-CD3 in combination with anti-CD28 antibodies at indicated time points at 37 °C. Cells were probed with antibodies to phospho-Zap-70 (Syk), Syk, phospo-PLCγ-1, PLCγ-1, phospho-Erk (p-p44/42 MAPK), Erk (p44/42), and GAPDH (*n* = 5–6). **c** Immunofluorescence of cultured CD8^+^ T cells from naïve P14 × WT mice; cells were activated (first line) or exhausted (second, third, and fourth lines) with GP33 peptide for 5 days at 37 °C. After 5 days, cells were cultured with GP33-loaded bone marrow-derived WT dendritic cells (DCs) for 30 min at 37 °C. One group of exhausted CD8^+^ T cells was additionally cultured with GP33-loaded bone marrow-derived WT DCs in the presence of isotype immunoglobulin G (IgG) monoclonal antibody (mAb; 40 µg ml^–1^, second line) or T cell receptor (TCR)-independent stimuli such as anti-CD44 mAb (40 µg ml^–1^; third line) or anti-CEACAM mAb (40 µg ml^–1^; clone CC1, last line) for 30 min at 37 °C. Cells were stained for Lck (red), filamin A (green), and DAPI (blue). Scale bars, 3 µm. *P *<* 0.05; **P *<* 0.01 (Student’s *t* test). ns = not significant. One representative blot of five to six (**b**) blots and four histology images of 4 to 6 (**a**, **c**) experiments are shown (mean ± SEM; **b**). Immunoblot images have been cropped for purposes of presentation. Full-sized images are presented in Supplementary Fig. 5

To test this hypothesis, we incubated GP33-loaded bone marrow-derived dendritic cells (BMDCs) from WT mice with GP33-specific (P14) naïve CD8^+^ T cells from P14 × WT or P14 × *Ceacam1*^*–/–*^ mice. Only GP33-specific P14 × WT CD8^+^ T cells formed an immunological synapse with DCs (Fig. 4a), whereas P14 × *Ceacam1*^*–/–*^ CD8^+^ T cells interacted with DCs but failed to recruit Lck and filamin A into the TCR signaling complex (Fig. 4a). To determine whether reduced Lck recruitment was associated with reduced TCR signaling, we purified splenic T cells from WT and *Ceacam1*^*–/–*^ mice and activated them with anti-CD3 in combination with anti-CD28 antibodies. *Ceacam1*^*–/–*^ T cells exhibited limited phosphorylation of Zap-70, PLCγ-1, and Erk44/42 (Fig. 4b), a finding suggesting that CEACAM1 indeed acts directly on Lck upstream of Zap-70 in the TCR activation pathway. This reduced phosphorylation of Zap-70, PLCγ-1, and Erk44/42 in the absence of CEACAM1 still allows initial proliferation of CD8^+^ T cells but results in reduced survival of proliferated CD8^+^ T cells after antigen activation (Fig. 3a, b, d).

Next, we determined whether Lck recruitment is affected in exhausted CD8^+^ T cells and whether we could restore its function by treatment with CEACAM1 antibodies. We hypothesized that treatment with anti-CEACAM1 antibody stabilizes CEACAM1, filamin A, and Lck within the TCR signaling complex. To test this idea, we cultured purified P14 × WT CD8^+^ T cells in the presence of activating and exhausting doses of GP33 peptide. Higher antigen load leads to deterioration of CD8^+^ T cell functions, which in turn leads to the expression of various inhibitory receptors on the surface of CD8^+^ T cells^7,45–48^. Compared to P14 × WT CD8^+^ T cells that received an activating dose of GP33 peptide, cultured P14 × WT CD8^+^ T cells that received an exhausting dose of GP33 peptide exhibited higher expression of inhibitory receptors, such as PD-1, lymphocyte-activating gene 3 (LAG-3) protein, and TIM3 (Supplementary Fig. 6a), and also exhibited reduced function in terms of the production of IFN-γ and TNF (Supplementary Fig. 6b) after in vitro restimulation with GP33 peptide. CD8^+^ T cells from both groups also stained positive for the expression of CD44 on their surface (Supplementary Fig. 6a).

Activated or exhausted CD8^+^ T cells were incubated with GP33-loaded DCs on day 5. Activated CD8^+^ T cells recruited Lck and filamin A into the immunological synapse (Fig. 4c, first line). In contrast, exhausted CD8^+^ T cells that received an isotype immunoglobulin G (IgG) monoclonal antibody (mAb) (Fig. 4c, second line) and a TCR-independent stimulus, such as anti-CD44 mAb (Fig. 4c, third line), when incubated with GP33-loaded DCs failed to recruit Lck into the immunological synapse. On the other hand, when anti-CEACAM1 mAb (clone CC1) was added to exhausted CD8^+^ T cells when incubated with GP33-loaded DCs, the recruitment of Lck into the immunological synapse was restored (Fig. 4c, fourth line). These findings show that CEACAM1 is essential for recruiting Lck into the TCR signaling complex to form an immunological synapse and that TCR signaling is enhanced during treatment with an anti-CEACAM1 mAb.

### Anti-CEACAM1 mAb treatment enhances CD8^+^ T cell function

**Fig. 5:**
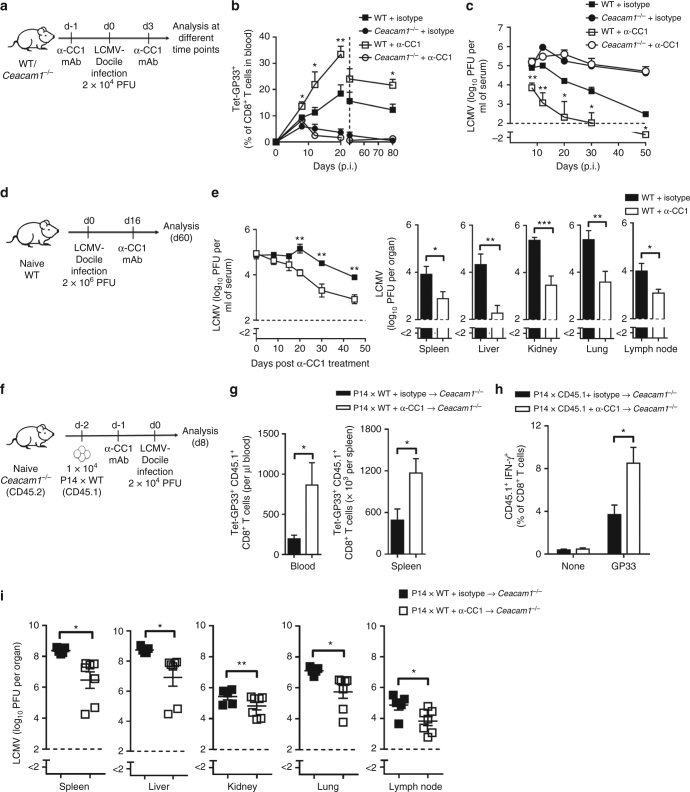
Anti-CEACAM1 mAb treatment enhances CD8^+^ T cell expansion and viral control. **a** Experimental setup. **b**, **c**. One group of wild-type (WT) and *Ceacam1*^*–/–*^ mice received anti-CEACAM1 mAb, and another group received an equal amount of isotype antibody on day –1 and day 3, followed by infection with 2 × 10^4^ PFU of LCMV-Docile on day 0. **b**, **c** Graphs showing percentage of virus-specific Tet-GP33^+^ CD8^+^ T cells in blood (**b**) and viral titers in serum (**c**) in indicated groups over the indicated times. Statistical significance is shown between the WT + IgG-treated groups and the WT + α-CC1 mAb-treated groups (*n* = 3–6 per group). **d** Experimental setup. **e** Wild-type (WT) mice were infected with 2 × 10^6^ PFU of LCMV-Docile on day 0. On day 16 after infection, mice were treated with 200 µg anti-CEACAM1 mAb per mouse or with an isotype antibody. **e** Viral titers in serum (left panel) over the indicated times and in indicated organs (right panel) on day 60 after infection (*n* = 4–8 per group). **f** Experimental setup. **g**–**i** *Ceacam1*^*–/–*^ mice were given 1 × 10^4^ P14 × CD45.1 WT splenocytes on day –2. One group of these mice was additionally treated with anti-CEACAM1 mAb, and another group was treated with an equal amount of an isotype antibody on day –1 and on day 3, followed by infection with 2 × 10^4^ PFU of LCMV-Docile on day 0. Mice were analyzed on day 8 after infection. **g** Absolute number of transferred Tet-GP33^+^ CD45.1^+^ CD8^+^ T cells in blood and spleen of host *Ceacam1*^*–/–*^ (CD45.2) mice (*n* = 5–7 per group). **h** Intracellular cytokine IFN-γ secretion from transferred P14 × CD45.1 WT CD8^+^ T cells (*n* = 5–7 per group). **i** Viral titers in the indicated organs of the recipient mice (*n* = 5–7 per group). *P *<* 0.05, **P *<* 0.01, ***P *<* 0.001 (Student’s *t* test). ns = not significant. Data are representative of two (**b**, **c**, **e**, **g**–**i**) independent experiments (mean ± SEM; **b, c, e, g**–**i**)

Next, we examined whether treatment with anti-CEACAM1 mAb could modulate the function of virus-specific CD8^+^ T cells in vivo. To do this, we injected WT and *Ceacam1*^*–/–*^ mice with an isotype control mAb or with anti-CEACAM1 mAb (clone CC1) and infected them with 2 × 10^4^ PFU of LCMV-Docile (Fig. 5a). Treatment with anti-CEACAM1 mAb increased the number of virus-specific CD8^+^ T cells, as measured by GP33-specific tetramer (Fig. 5b); because of these enhanced numbers, viral infection was resolved more rapidly (Fig. 5c). Efficient CD8^+^ T cell function and more rapid clearance of virus caused less damage to liver hepatocytes and resulted in reduced immunopathology, as measured by the activity of the liver-specific enzymes alanine transaminase (ALT) and aspartate transaminase (AST) in mice treated with anti-CEACAM1 mAb (Supplementary Fig. 7).

Next, we investigated whether anti-CEACAM1 mAb treatment can be used therapeutically during an ongoing viral infection. We infected WT mice with LCMV-Docile, thereby establishing a chronic viral infection in those mice. On day 16 after infection, one group of mice was treated with an isotype control mAb, whereas a separate group of mice was treated with anti-CEACAM1 mAb (Fig. 5d). Mice treated with anti-CEACAM1 mAb exhibited lower viral loads than did mice treated with isotype mAb (Fig. 5e). To further determine whether treatment with anti-CEACAM1 mAb acts specifically on CD8^+^ T cells, we transferred equal number of P14 × WT (CD45.1) splenocytes into *Ceacam1*^*–/–*^ mice and then treated those mice with anti-CEACAM1 mAb or isotype mAb, followed by LCMV-Docile infection (Fig. 5f). We found that treatment with anti-CEACAM1 mAb enhances virus-specific CD8^+^ T cell responses not only numerically (Fig. 5g) but also functionally by their ability to produce IFN-γ (Fig. 5h). Viral titers were significantly reduced in several organs by treatment with anti-CEACAM1 mAb 8 days after infection (Fig. 5i). Together, these findings suggest that treatment with anti-CEACAM1 antibody enhances the function of CD8^+^ T cells not only quantitatively but also qualitatively and thereby restores the function of exhausted CD8^+^ T cells in chronic viral infection.

### Anti-hCEACAM1 mAb enhances T cell functions of human cells

**Fig. 6:**
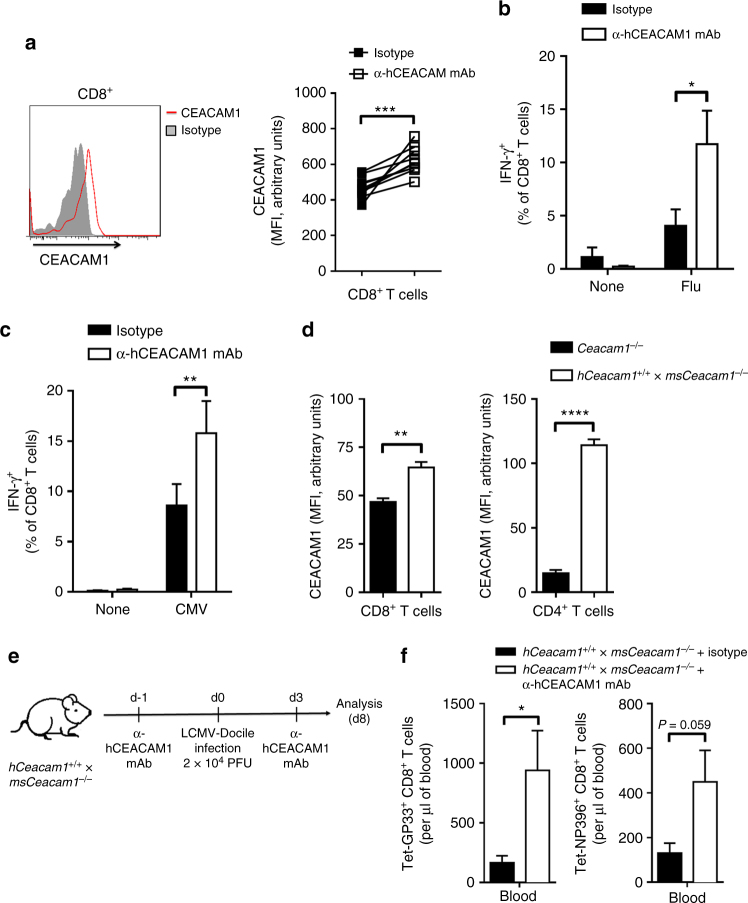
Treatment with anti-hCEACAM1 mAb improves CD8^+^ T cell function in human cells. **a** Representative fluorescence-activated cell sorting (FACS) histogram (left panel) showing CEACAM1 expression and mean fluorescence intensity (MFI) levels (right panel) of naïve CD8^+^ T cells from the peripheral blood of healthy donors (*n* = 10). **b**, **c** Intracellular cytokine (interferon-γ (IFN-γ)) secretion by peripheral blood mononuclear cells (PBMCs) from healthy donors that were cultured with Flu peptide together with anti-CD28 antibody (**b**) or with or without cytomegalovirus (CMV) peptide (**c**) and human interleukin-2 (IL-2) in the presence or absence of anti-hCEACAM1 antibody (40 µg ml^–1^; clone 18/20) for 10 days. Cytokine production was assessed by restimulation of PBMCs with or without Flu peptide (**b**, *n* = 8–10 per group) for 6 h or with CMV peptide (**c**; *n* = 9 per group) for 8 h at 37 °C, as measured with flow cytometry. **d** Histogram showing mean fluorescence intensity (MFI) levels of CEACAM1 expression in peripheral blood CD8^+^ (left panel) and CD4^+^ (right panel) T cells from *hCeacam1*^+/+^ × *msCeacam1*^*–/–*^ mice as measured by flow cytometry (*n* = 4–5 per group). **e** Experimental setup. Human CEACAM1 transgenic mice (*hCeacam1*^+/+^ × *msCeacam1*^*–/–*^) were injected with anti-hCEACAM1 antibody (clone 18/20) or isotype antibody on day –1 (100 µg/mouse) and day 3 (200 µg/mouse), followed by infection with 2 × 10^4^ PFU of LCMV-Docile on day 0. **f** Number of virus-specific Tet-GP33^+^ (left panel) and NP396^+^ (right panel) CD8^+^ T cells in blood from *hCeacam1*^*+/+*^ × *msCeacam1*^*–/–*^ mice on day 8 after infection (*n* = 5 per group). *P *<* 0.05, **P *<* 0.01, ***P *<* 0.001; ****P *<* 0.0001 (Student’s *t* test). Data are representative of two (**a**, **d**, **f**) or three (**b**, **c**) experiments (mean ± SEM; **a**–**d**, **f**)

Next, we determined whether anti-CEACAM1 mAb could be used to enhance the function of virus-specific CD8^+^ T cells in human peripheral blood mononuclear cells (PBMCs). We tested the binding of human CEACAM1 mAb (clone 18/20, made in-house) on CD8^+^ T cells from healthy donors. Anti-human CEACAM1 (anti-hCEACAM1) antibody exhibited binding to CD8^+^ T cells (Fig. 6a). To determine whether this antibody could enhance CD8^+^ T cell function, we cultured PBMCs from healthy donors with anti-CD28 antibody in the presence of influenza (Flu) peptide (GILGFVFTL) with or without anti-hCEACAM1 mAb. We found that anti-hCEACAM1 mAb enhanced the production of IFN-γ by CD8^+^ T cells after restimulation with the Flu peptide (Fig. 6b). Similarly, PBMCs from healthy donors exhibited higher IFN-γ production when cultured with cytomegalovirus (CMV) peptide (NLVPMVATV) in the presence of anti-hCEACAM1 mAb (Fig. 6c). To determine whether anti-hCEACAM1 mAb can also improve the function of CD8^+^ T cells in vivo, we used a mouse model expressing human CEACAM1 as a transgene. Newly generated naïve *hCeacam1*^*+/+*^ × *msCeacam1*^*–/–*^ mice exhibited a splenic structure resembling that of naïve WT mice (Fig. 2) and expressed substantial levels of CEACAM1 on peripheral blood T cells (Fig. 6d). We treated *hCeacam1*^*+/+*^ × *msCeacam1*^*–/–*^ mice with anti-hCEACAM1 antibody and then infected them with LCMV-Docile (Fig. 6e). Treatment with anti-hCEACAM1 mAb enhanced virus-specific CD8^+^ T cell numbers (Fig. 6f). The number of GP33 tetramer-specific CD8^+^ T cells was significantly higher in anti-hCEACAM1 mAb-treated mice than isotype control mAb-treated mice. Moreover, the number of NP396 tetramer-specific CD8^+^ cells was slightly higher in mice treated with anti-hCEACAM1 mAb than in mice treated with isotype control mAb (Fig. 6f). Therefore, we conclude that treatment with anti-hCEACAM1 mAb improves virus-specific CD8^+^ T cell numbers and function both in vitro and in vivo.

## Discussion

In this report we describe the involvement of CEACAM1 in chronic viral infection. We found that lack of CEACAM1 was correlated with deficient control of LCMV. Certainly, reduced number of B cells and CD169^+^ marginal zone macrophages contribute to deficient control of LCMV. We then focused on the cell-intrinsic role of CEACAM1 in CD8^+^ T cells. The absence of CEACAM1 in CD8^+^ T cells resulted in insufficient CD8^+^ T cell functions. By conducting adaptive cell transfer experiments, we found that CD8^+^ T cell-intrinsic CEACAM1 deficiency resulted in reduced viral control in the spleen, lymph node, lung, liver, and serum. Therefore, the absence of CEACAM1 within CD8^+^ T cells contribute to lack of viral control in *Ceacam1*^*–/–*^ mice. Mechanistically, we found that CEACAM1 deficiency within CD8^+^ T cells was associated with limited TCR activation. CEACAM1 was involved in the recruitment of Lck into the immunological synapse and thereby promoted the phosphorylation of Zap-70, PLCγ-1, and Erk44/42. Additionally, the absence of CEACAM did not influence the proliferation of CD8^+^ T cells, but did affect the survival of proliferating CD8^+^ T cells after activation. In line with this finding, the generation of mature CD4^+^ and CD8^+^ T thymocytes was slightly reduced in the absence of CEACAM1. However, the difference is less pronounced than in the generation of virus-specific CD8^+^ T cells in the absence of CEACAM1.

CEACAM1 has very diverse functions in the immune system. CEACAM1 has been described primarily as a regulator of T cells in the gut^26,27,29,49–51^. The expression of CEACAM1-L inhibits T cell proliferation and thereby prevents inflammatory bowel disease, whereas the expression of CEACAM1-S is essential for the development of Tfh-driven IgA production by gut B cells^27^. Recently, we found that the expression of CEACAM1-L on B cells enhances Syk phosphorylation after B cell receptor activation and therefore is essential for B cell development, survival, and proliferation^17,18^. In line with these data, expression of CEACAM1 maintains the splenic architecture including the development of B cell follicles, and CD169^+^ metallophilic macrophages^18^. Both B cells and marginal zone macrophages play an important role during LCMV infection^37^^,^^52^. CD169^+^ macrophages, specifically, play an integral function in preventing early viral propagation immediately after infection^37,53^. Therefore, it is likely that in addition to reduced CD8^+^ T cell function, the reduced number of marginal zone macrophages contribute to defective LCMV control in *Ceacam1*^*–/–*^ mice.

Our current findings show that the expression of CEACAM1 on CD8^+^ T cells enhances TCR signaling in virus-specific CD8^+^ T cells via recruitment of Lck at the immunological synapse. In accordance with Klaile et al.^33^, we are convinced that interaction with the long isoform of CEACAM1 (CEACAM1-4L) on exhausted CD8^+^ T cells stabilizes Lck activity and restores exhausted CD8^+^ T cells. Additionally, binding of anti-CEACAM1 mAb to various extracellular domains of CEACAM1 can fine-tune the activatory signals upon ligation. Therefore, we identified CEACAM1 as an important player in the activation of CD8^+^ T cells.

During persistent viral infection, treatment with anti-CEACAM1 antibody enhanced LCMV-specific CD8^+^ T cell expansion and proliferation and also controlled chronic infection within 3 weeks, whereas chronic infection persisted in control animals. Administration of an anti-hCEACAM1 antibody improved the number and the IFN-γ production of virus-specific CD8^+^ T cells both in vitro (with virus-specific human CTLs) and in vivo in a human CEACAM1 transgenic mouse model.

We propose that treating CD8^+^ T cells with anti-CEACAM1 antibody stabilizes Lck within the TCR signaling complex during LCMV infection. This mechanism could add to the recently described interaction between CEACAM1 and TIM3^32^: anti-CEACAM1 antibody blocks the interaction of TIM3 and CEACAM1 and thereby limits ITIM phosphorylation, resulting in prolonged TCR signaling^32^. Additionally, simultaneously blocking the TIM3 and PD-1 pathways restores CD8^+^ T cell function and results in better control of virus^54^. Whether this mechanism contributes to the activating effects found in the current study remains to be further investigated.

The current study found that, during chronic viral infection, CEACAM1 activation strongly improves the antiviral CD8^+^ T cell response and resolves viral infection. Also, virus-specific human CD8^+^ T cells can be strongly activated by treatment with a species-specific CEACAM1 antibody. Obviously, it remains unknown whether treatment with anti-CEACAM1 antibody for various types of viral infections, autoimmune diseases, and cancer could similarly benefit T cell function. On the basis of our findings, we speculate that whenever the recruitment of Lck into the immunological synapsis is limited, anti-CEACAM1 treatment would improve CD8^+^ T cell function and could improve the success of vaccination.

In conclusion, we found that CEACAM1 is an important player in resolving acute and chronic viral infections and that treatment of chronic viral infections with anti-CEACAM1 antibody enhances antiviral CD8^+^ T cell responses. Various effects on other immune components may also benefit from a CEACAM1-targeted therapeutic approach with respect to a more rapid control of viral infection. The use of an anti-CEACAM1 antibody to combat chronic viral infection appears to be a powerful therapeutic option.

## Methods

### Mice

Mice used in this study, including WT and *Ceacam1*^*–/–*^ mice, were maintained on the C57BL/6 genetic background (back-crossed as many as 16 times) and were bred as homozygotes. *Ceacam1*^*–/–*^ mice were kindly provided by Prof. Nicole Beauchemin (Rosalind and Morris Goodman Cancer Centre, Montreal, QC, Canada)^23^. Human CEACAM1 transgenic mice were originally developed and were kindly provided by Prof. J. Shively (Beckman Research Institute at City of Hope, Duarte, CA, USA)^55^. They were back-crossed eight times into the C57BL/6 background and then crossed eight times with C57BL/6 *Ceacam1*^*–/–*^ mice to generate C57BL/6-*hCEACAM1*^+/+^ *×* *msCeacam1*^*–/–*^ mice (established by one of the authors (B. B. S.)). For adoptive transfer experiments, mice congenic for CD45 (CD45.1 or CD45.2) were used to distinguish between transferred cells (CD45.1 or CD45.2) and endogenous (CD45.1 or CD45.2) cells. P14 × D45.2 WT, P14 × *Ceacam1*^*–/–*^ (CD45.2), or P14 × CD45.1 WT mice expressing an LCMV-GP33-specific T cell receptor as a transgene were used for cell transfer studies.

Six-week-old to eight-week-old age-matched and sex-matched mice were used for all experiments. All animals were housed in single ventilated cages. Animal experiments were authorized by the Nordrhein Westfalen Landesamt für Natur, Umwelt und Verbraucherschutz (Recklinghausen, Germany) and in accordance with the German law for animal protection.

### Virus and plaque assay

LCMV-WE was originally obtained from F. Lehmann-Grube (Heinrich Pette Institute, Hamburg, Germany) and was propagated on L929 cells (obtained from ATCC, NCTC clone 929). LCMV-Docile was donated by Prof. Dr. R. Zinkernagel (University of Zurich, Zurich, Switzerland) and was propagated on L929 cells. Mice were infected intravenously with either 200, 2 × 10^4^, or 2 × 10^6^ PFU of LCMV-Docile or LCMV-WE. LCMV viral titers were detected by a plaque-forming assay on MC57 fibroblasts, as previously described^18,56^. In brief, organs were smashed in Dulbecco’s modified Eagle’s medium (DMEM) containing 2% fetal calf serum (FCS), titrated 1:3 over 12 steps in 96-well round-bottom plates, and plaqued on MC57 cells. After incubation for 3 h at 37 °C, the overlay was added (1:1 mixture of methyl cellulose and Iscove’s modified Dulbecco’s medium), and the virus preparation was again incubated at 37 °C. Plaques were counted 48 h later by LCMV nucleoprotein (NP) staining. For staining, cells were fixed with 4% formaldehyde solution in phosphate-buffered saline (PBS), permeabilized with 1% Triton-X solution in PBS, blocked with 10% FCS in PBS, and stained with rabbit anti-LCMV NP antibody (made in-house). Enhanced chemiluminescence-conjugated anti-rabbit-IgG antibody (from Jackson ImmunoResearch) was used as a secondary antibody (diluted 1:400 to the original concentration in distilled water). Plaques were detected by chemiluminescence (0.2 M Na_2_HPO_4_ + 0.1 M citric acid + 30% H_2_O_2_ + *o*-phenylenediamine dihydrochloride; all chemicals from Sigma-Aldrich).

### Flow cytometry

Tetramers were provided by the Tetramer Facility of the National Institutes of Health (NIH; Bethesda, MD, USA). Cells were stained with allophycocyanin-labeled GP33 (GP33/H-2Db) and NP396 MHC class I tetramers for 15 min at 37 °C. After incubation, the samples were stained with anti-CD8 (53–6.7) (eBioscience) or anti-CEACAM1 antibody (clone CC1, a kind gift from Dr. Kathryn V. Holmes, University of Colorado, Denver, CO, USA) for 30 min at 4 °C. For blood samples, erythrocytes were lysed with 1 ml BD lysing solution (BD Biosciences), washed once with fluorescence-activated cell sorting (FACS) buffer, and analyzed by flow cytometry. Absolute number of GP33-specific and NP396-specific CD8^+^ T cells were calculated by FACS analysis using fluorescing beads (BD Biosciences). For measurement of intracellular IFN-γ and TNF, cells were fixed with formaldehyde (2% formaldehyde solution in PBS) for 10 min, permeabilized with saponin (1%) solution, and stained with anti-IFN-γ (XMG 1.1) or TNF (MP6-XT22) antibodies (both from eBioscience).

For splenocyte transfer experiments, GP33^+^ CD8^+^ T cells were stained as described above; in addition, cells were stained with anti-CD8 (53-6.7), anti-CD45.1 (A20), and anti-CD45.2 (104) antibodies (all from eBioscience). Human peripheral blood cells were stained with anti-CD8 (SK1) (eBioscience) and anti-hCEACAM1 (clone 18/20, provided by one of the authors (B.B.S.), made and labeled in-house) or anti-hCEACAM1 (CD66a-B1.1, eBioscience) antibodies, with corresponding isotype anti-IgG1 (labeled in-house) and anti-IFN-γ (4s.B3, eBioscience) antibodies. To check the expression of surface markers, cultured cells were stained with anti-PD-1 (RMP1-30, Invitrogen), anti-LAG-3 (ebioC9B7W, eBioscience), anti-TIM3 (RMT3-23, BioLegend), and anti-CD44 (Pgp-1, Ly-24, eBioscience) antibodies. All antibodies were diluted 1:100 to their original concentration in FACS buffer. For determination of total cell numbers, FACS beads were used (BD Biosciences). All stained cells were analyzed on an LSR II or a FACS Fortessa flow cytometer (BD Biosciences), and data were analyzed with the FlowJo software (FlowJo LLC, Ashland, OR, USA).

### Intracellular cytokine staining

For intracellular cytokine staining (ICS), splenic tissue was homogenized, and splenocytes were restimulated with LCMV-GP33-specific peptide (PolyPeptide Laboratories, Strasbourg, France) for 6 h. Brefeldin A (BFA) was added 1 h after stimulation, and samples were incubated at 37 °C, after which they were incubated with surface and intracellular markers at 4 °C, as described above. Human PBMCs were cultured for 10 days in media containing 50 IU ml^−1^ IL-2 (Roche) and 1 µg ml^−1^ peptide (Flu, GILGFVFTL; CMV, NLVPMVATV; IBA Lifesciences, Goettingen, Germany) in the presence or absence of anti-hCEACAM1 antibody (40 µg ml^−1^, clone 18/20). After 10 days, cells were restimulated with Flu or CMV peptide in the presence of BFA for 6 h, after which they were subjected to various types of staining for surface and intracellular markers, as described above. Human PBMCs collected from healthy donors were processed from buffy coats obtained at the center for blood donation from the University Hospital Düsseldorf. Written informed consent was obtained from all participants and the study was approved by the ethics committee of the University Hospital Düsseldorf (study number 3240). These samples were anonymized, and no additional donor information was available. Cell surface staining to determine CTL activity was performed with BD reagents as described^57^. In short, T cell antibodies, anti-CD107a (1D4B), anti-CD43 (1B11), and anti-CD62L (MEL-14, all from BD Biosciences) were used for surface staining. Dead cells (propidium iodide-positive) were excluded from all cell surface analysis. Intracellular granzyme B (GB12, Caltag Laboratories) staining was performed with the Cytofix/Cytoperm Intracellular Staining Kit (BD Biosciences) after a 4-h stimulation of splenocytes with anti-CD3e (500A2) and anti-CD28 (37.51, both from BD Biosciences) antibodies.

### Splenocyte/lymphocyte transfer

As described above, 1 × 10^4^ splenocytes from P14 × WT and P14 × *Ceacam1*^*–/–*^ mice expressing CD45.2 as a congenic marker were injected intravenously into CD45.1 (WT) mice or P14 × CD45.1 WT into CD45. 2 (*Ceacam1*^*–/–*^) mice. One day later, mice were infected with 2 × 10^4^ PFU of LCMV-Docile. The percentage or an actual number of proliferated P14 CD8^+^ T cells was assessed in various compartments by flow cytometry 8 days after infection. The purity of sorted cells was maintained at higher than 95% for the transfer. For cell proliferation assays, cells were labeled with 5 µM CFSE, and 1 × 10^7^ splenocytes per mouse were transferred into recipient mice. The proliferation was assessed by flow cytometry, and data were analyzed with FlowJo software.

### Bone marrow chimeras

For the generation of bone marrow chimeras, C57BL/6 (WT) mice were irradiated with a total of 1050 rad. After 24 h, the immune system of the mice was reconstituted intravenously with 5 × 10^6^ bone marrow cells from each donor for mixed bone marrow chimeras. Mice were analyzed 45 days after reconstitution.

### Immunoblotting

Spleens were dissociated, and T cells were purified with Pan T Cell Isolation Kit (magnetic-activated cell sorting (MACS), Miltenyi Biotech) according to standard techniques and with MACS columns. Next, 2 × 10^6^ cells were challenged with or without anti-CD3e (500A2, 4 µg ml^−1^) in combination with anti-CD28 (37.51, 2 µg ml^−1^) antibodies (both from BD Biosciences) for various time periods. Cells were lysed in boiling sodium dodecyl sulfate (SDS) buffer (1.1% SDS, 11% glycerol, 0.1 M Tris; pH 6.8) with 10% 2-mercaptoethanol (all from Sigma). Total cell extracts were examined with 10% SDS-polyacrylamide gel electrophoresis gels and transferred onto Whatman nitrocellulose membranes (GE Healthcare) according to standard techniques. Membranes were blocked for 1 h in 5% bovine serum albumin (BSA; PAA Laboratories) in PBS supplemented with 1% Tween-20 and were incubated with the following antibodies: anti-phospho-Zap-70 (Syk) (Y352/Y319); anti-phospho-PLCγ-1; anti-phospho-p44/42 (p-Erk1/2); anti-Syk; anti-PLCγ-1; and anti-p44/42 (Erk1/2) (all from Cell Signalling Technology). The secondary antibody anti-GAPDH (Meridian Life Science) was detected by horseradish peroxidase-conjugated anti-mouse IgG antibody (Bio-Rad Laboratories), anti-rabbit IgG antibody (GE Healthcare), or both. Signals were detected with the Bio-Rad ChemiDoc imaging system (Bio-Rad Laboratories) and analyzed with the manufacturer’s software. Images have been cropped for purposes of presentation.

### Antibody injections

We intravenously injected 100 or 200 µg of anti-mouse CEACAM1 antibody (clone CC1), anti-hCEACAM1 antibody (clone 18/20), or an isotype control mAb into each mouse at day −1 and day 3 of infection, respectively.

### Generation of BMDCs

Primary DCs were generated by isolating bone marrow cells from femurs and tibias of mice, followed by the elimination of erythrocytes. Bone marrow cells were cultured in very low endotoxin-DMEM (Biochrom) supplemented with 10% (v v^−1^) lipopolysaccharide (LPS)-free FCS (Biochrom); 1% (v v^−1^) penicillin-streptomycin-glutamine (Thermo Fisher Scientific); 0.1% (v v^−1^) 50 mM β-mercaptoethanol (β-ME) (Invitrogen); and 10 ng ml^−1^ granulocyte–macrophage colony-stimulating factor (made in-house). Cells were treated with lipopolysaccharide (100 ng ml^−1^, Sigma-Aldrich) for 24 h on day 6. On day 7 after harvesting, cells were washed with Dulbecco’s phosphate-buffered saline (DPBS), and 5 × 10^5^ cells were transferred into a 24-well plate containing coverslips coated with poly-l-lysine (Sigma-Aldrich).

### Confocal microscopy

CD8^+^ T cells were purified with the CD8^+^ T Cell Isolation Kit (MACS; Miltenyi Biotech) according to standard techniques: spleens were homogenized in MACS buffer (1% FCS and 0.8% 0.5 M ethylenediaminetetraacetic acid in DPBS). For histologic analysis, coverslips were coated with poly-l-lysine solution (Sigma-Aldrich) for 10 min before DCs were added. Cells were loaded with GP33 peptide (1 µg ml^–1^) by incubation for 2 h at 37 °C with intermittent shaking. Cells were then washed with DPBS, after which an equal number of purified CD8^+^ T cells were added at 37 °C. Purified CD8^+^ T cells were cultured in Iscove’s modified Dulbecco’s medium (Lonza) supplemented with 10% (v v^−1^) LPS-free FCS (Biochrom); 1% (v v^−1^) penicillin-streptomycin (Thermo Fisher Scientific); and 0.1% (v v^−1^) 50 mM β-ME (Invitrogen). After 30 min of incubation, cells were washed with DPBS and were then fixed (with 2% formaldehyde in DPBS) and permeabilized (with 0.1% Triton-X in DPBS). Nonspecific antigens were blocked with 2% BSA in DPBS for 15 min. Cells were then stained with anti-Lck (3A5, sc-433; Santa Cruz Biotechnology), anti-filamin A (ab11074; Abcam), and DAPI (4′,6-diamidino-2-phenylindole, Invitrogen) antibodies. Fluorescently labeled anti-mouse (Thermo Fisher Scientific) and anti-goat (Invitrogen) secondary antibodies were used to detect the signals. Antibodies were diluted 1:250 to their original concentration in blocking solution and incubated for 45 min at room temperature.

For in vitro activation and exhaustion of T cells, purified CD8^+^ T cells were cultured in media as described above. Next, 1 × 10^6^ P14 CD8^+^ T cells were activated by the addition of GP33 peptide (1 µg ml^–1^) on day 0 and were exhausted by the addition of GP33 peptide (1 µg ml^–1^) to the culture media on days 0, 1, 2, 3, 4, and 5. Isotype IgG (40 µg ml^–1^), anti-CD44 (40 µg ml^–1^, clone IM7; BioLegend), and anti-CEACAM1 (40 µg ml^–1^, clone CC1) mAbs were added for 30 min, along with activated and exhausted P14 CD8^+^ T cells. Images of stained sections were acquired with a confocal microscope (Leica SP8 gSTED; Leica Microsystems) with a ×40 zoom objective. Imaris software (Bitplane AG, Zurich, Switzerland) was used for 3D reconstruction.

### Histology

Histologic analyses of snap-frozen tissue were performed with a mAb to anti-CD45R (B220; RA3-6B2) and anti-CD90.2 (53-2.1) (both from eBioscience). Biotin anti-CD169/SIGLEC1 (MOMA-1) (Acris) antibody was used to stain CD169 macrophages. Sections were washed and stained with streptavidin antibody (eBioscience). In short, sections were fixed with acetone for 10 min, and nonspecific antigen binding was blocked in PBS containing 2% FCS for 15 min, followed by staining with various antibodies for 45 min. All antibodies were diluted 1:100 from their original concentration in blocking solution. Images of stained sections were acquired with a fluorescence microscope (KEYENCE BZ II analyzer; KEYENCE Corporation of America, Itasca, IL, USA).

### Alanine transaminase and aspartate transaminase

The activity of ALT and AST was measured at the Central Laboratory, University Hospital Essen, Germany.

### Statistical analysis

Data are expressed as means ± SEM from independent experiments. Student’s *t* test was used to detect statistically significant differences between groups. No sample or value was excluded from the analysis. The level of statistical significance was set at *P* *<* 0.05. Graphs were prepared with GraphPad Prism 6 (GraphPad Software, La Jolla, CA, USA).

### Data availability

All data supporting the findings of this study are available from the corresponding authors upon request.

## Electronic supplementary material

Supplementary Information
