# Supplementary material for: CEACAM1 promotes CD8+ T cell responses and improves control of a chronic viral infection
Source: Nat Commun. 2018 Jul 2;9:2561. doi: 10.1038/s41467-018-04832-2 (PMC6028648; doi:10.1038/s41467-018-04832-2)
Supplement: Supplementary file 1 — Supplementary Information [file 41467_2018_4832_MOESM1_ESM.pdf]

CEACAM1 promotes CD8$^+$ T-cell responses and improves control of a chronic viral infection

Khairnar et al.

Supplemental Figure

Supplementary Fig. 1

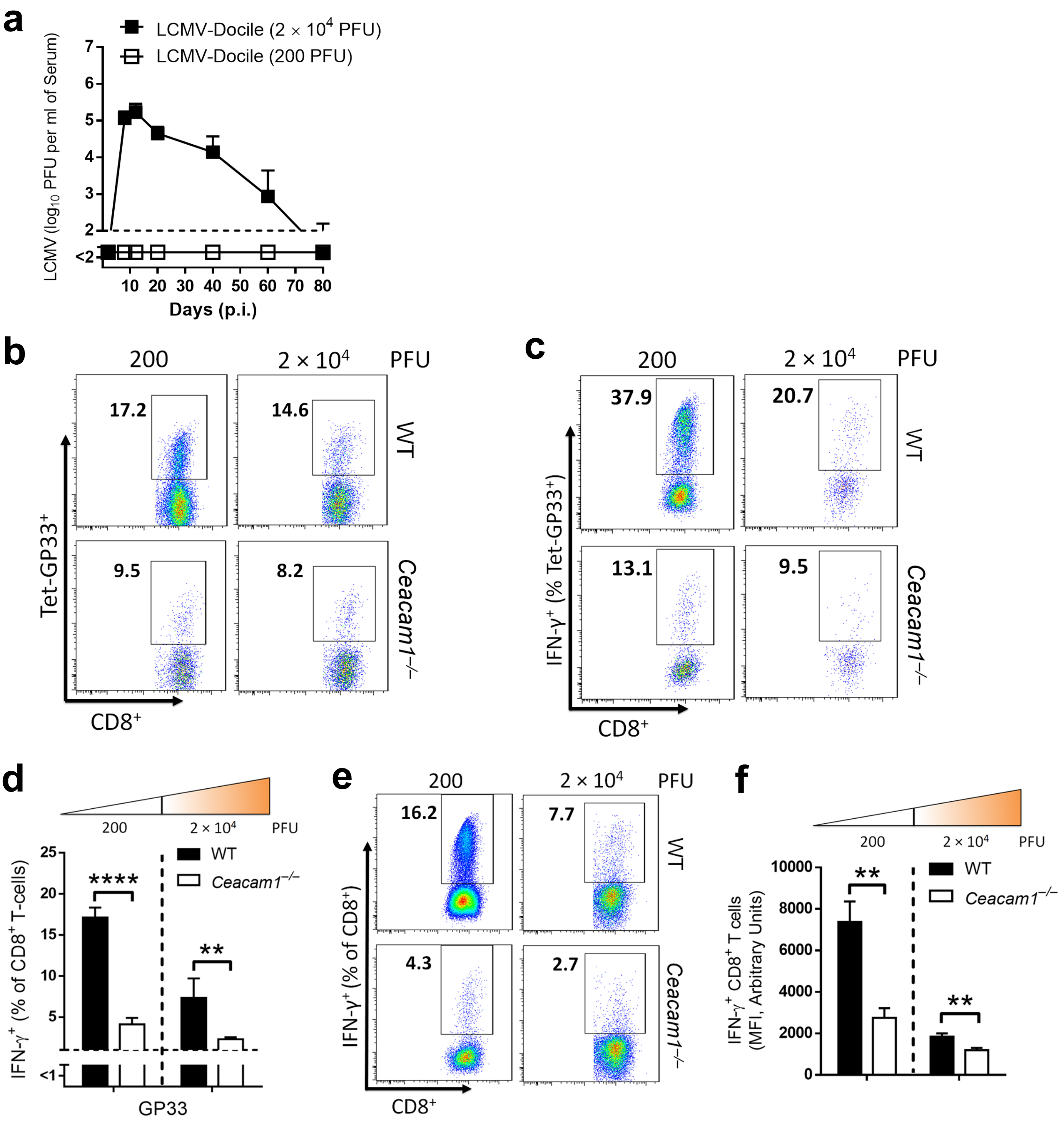

Supplementary Figure 1. CEACAM1 is important for CD8+ T-cell function after infection with LCMV. **a:** Viral titers in serum from wild-type (WT) mice on indicated days after intravenous infection with 200 or 2×10^4 PFU of LCMV-Docile (n = 5-8 per group). **b-c:** Representative FACS plots showing the percentage of virus-specific Tet-GP33+ CD8+ T cells in blood (**b**) or the secretion of intracellular cytokine interferon (IFN)-γ by virus-specific Tet-GP33+ CD8+ T cells in splenocytes (**c**) of wild-type (WT) and *Ceacam1*$^{-/-}$ mice 8 days after infection with 200 or 2×10^4 PFU of LCMV-Docile (n = 6-8 per group). **d-f:** Histogram (**d**) of representative FACS plots showing the percentage (**e**) and mean fluorescence intensity (MFI) levels (**f**) of intracellular cytokine interferon (IFN)-γ secretion from total CD8+ T cells by splenocytes of wild-type (WT) and *Ceacam1*$^{-/-}$ mice on day 8 after infection with 200 or 2×10^4 PFU of LCMV-Docile. Horizontal dotted lines designate the detection limit without restimulation (n = 6-8 per group). $**P < 0.01$; $****P < 0.0001$ (Student's t-test). Data are representative of two (**a-f**) experiments (mean ± SEM; **a**, **d**, & **f**).

Supplementary Fig. 2

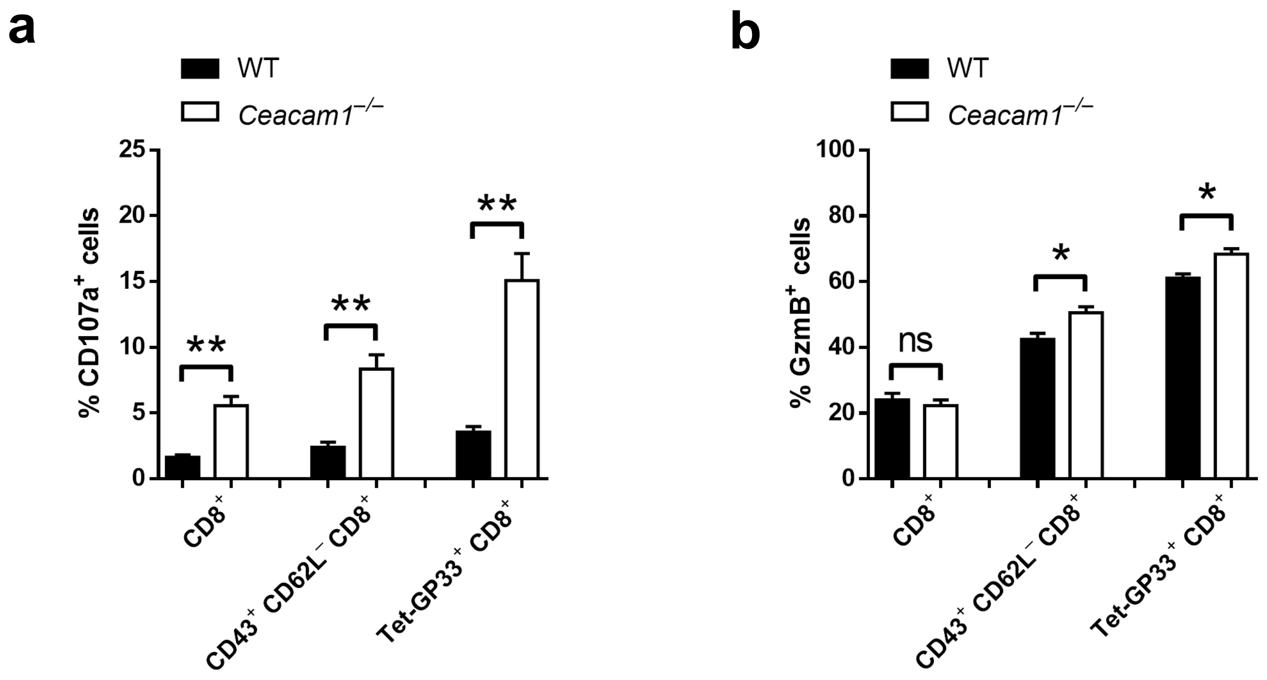

Supplementary Figure 2. ***Ceacam1*$^{-/-}$ mice exhibit higher cytotoxic T lymphocyte responses.**
a-b: Histograms showing the percentage of CD107a^{+} cells (**a**) and granzyme B expression (**b**) in CD8^{+} T cells, CD43^{+} CD62L^{-} CD8^{+} T cells, and virus-specific Tet-GP33^{+} CD8^{+} T cells from splenocytes of wild-type (WT) and *Ceacam1*$^{-/-}$ mice on day 8 after infection with 2×10^{4} PFU of LCMV-Docile (n = 4 per group). **P* < 0.05; ***P* < 0.01 (Student's t-test). ns = not significant. (mean ± SEM; **a** & **b**).

Supplementary Fig. 3

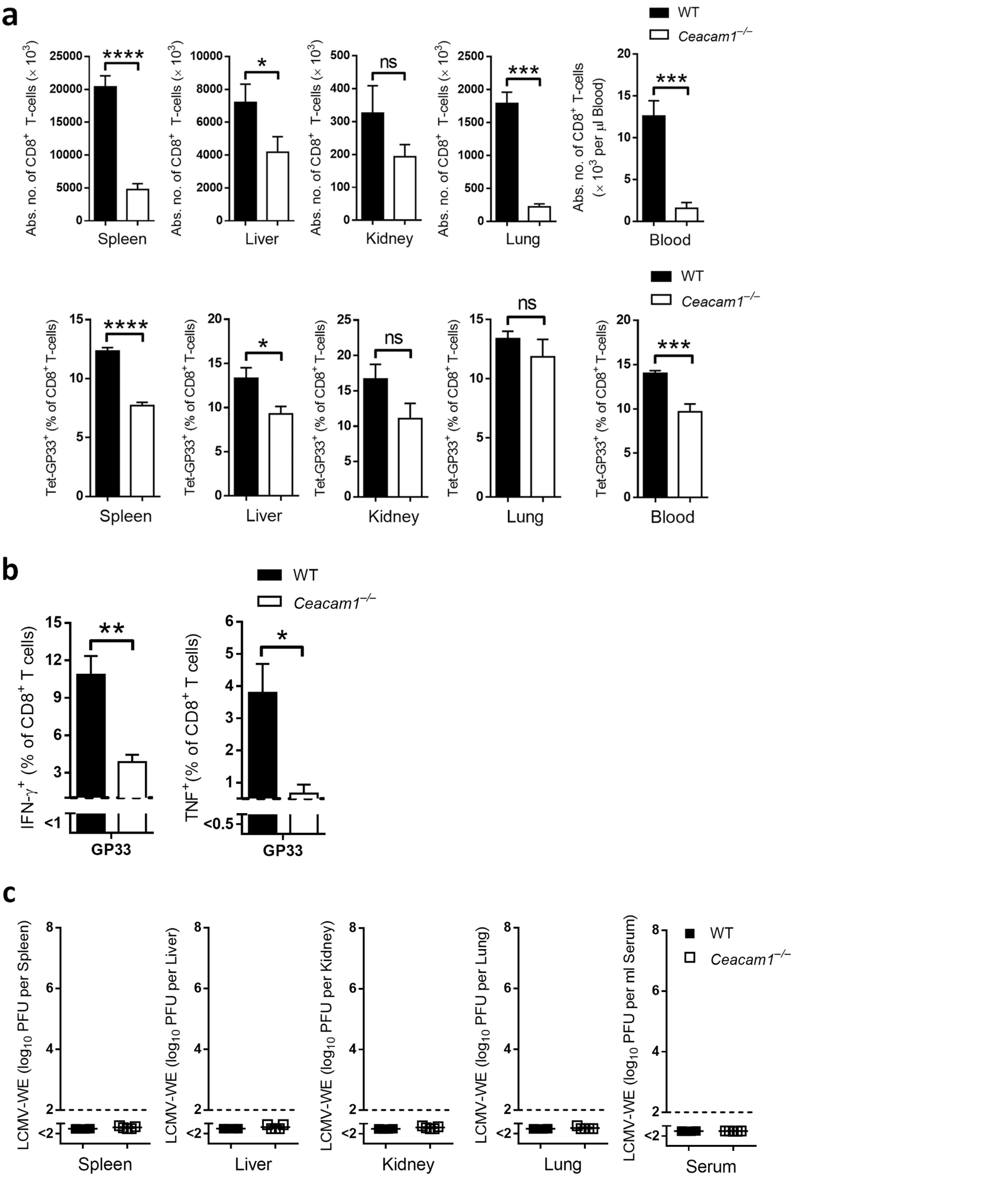

Supplementary Figure 3. CEACAM1 is important for CD8+ T-cell expansion and function in acute viral infection.

a-c: Total numbers of CD8+ T cells and percentages of virus-specific Tet-GP33+ CD8+ T cells in indicated organs and blood (**a**), intracellular cytokine interferon (IFN)-γ and tumor necrosis factor alfa (TNF) secretion in splenocytes (**b**), and viral titers in indicated organs and serum (**c**) from wild-type (WT) and *Ceacam1*−/− mice on day 8 after infection with 200 PFU of LCMV-WE. Horizontal dotted lines designate the detection limit for cytokines without restimulation (**b**) and viral titers (**c**) (n = 5 per group). $*P < 0.05$; $**P < 0.01$; $***P < 0.001$; $****P < 0.0001$ (Student's t-test). ns = not significant. Data are representative of two (**a, b** & **c**) experiments (mean ± SEM; **a**, **b**).

Supplementary Fig. 4

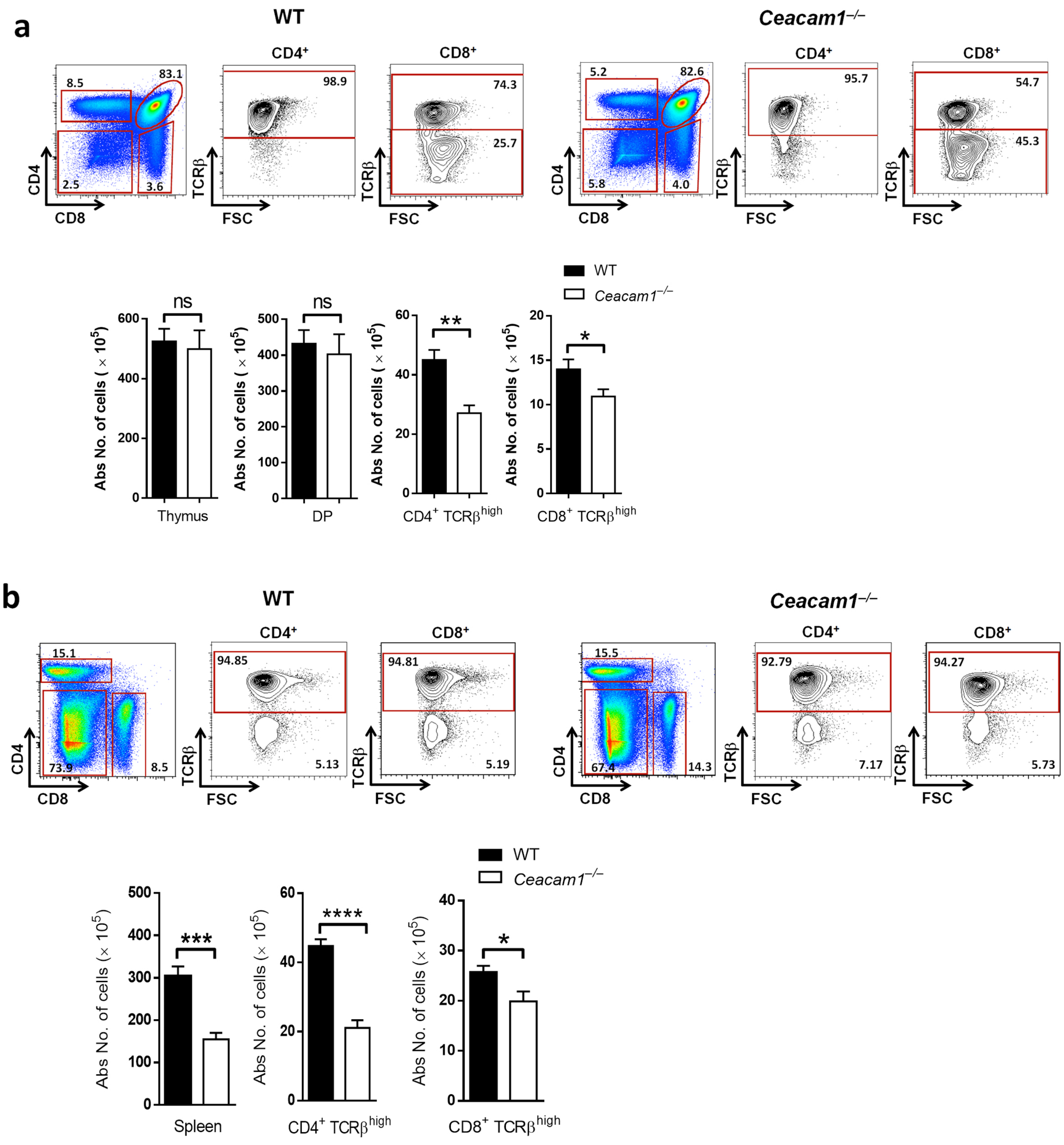

Supplementary Figure 4. CEACAM1 affects T-cell development *in vivo*.
a-b: Representative FACS plots, gating strategy, and absolute numbers of T-cell subpopulations in thymus (**a**; n = 5-6 per group) and spleen (**b**; n = 5-6 per group) of wild-type (WT) and *Ceacam1*$^{-/-}$ mice as measured by flow cytometry. *$P < 0.05$; **$P < 0.01$; ***$P < 0.001$; ****$P < 0.0001$ (Student's t-test). ns = not significant. Data are representative of two (**a** & **b**) experiments (mean ± SEM; **a**, **b**).

Supplementary Fig. 5

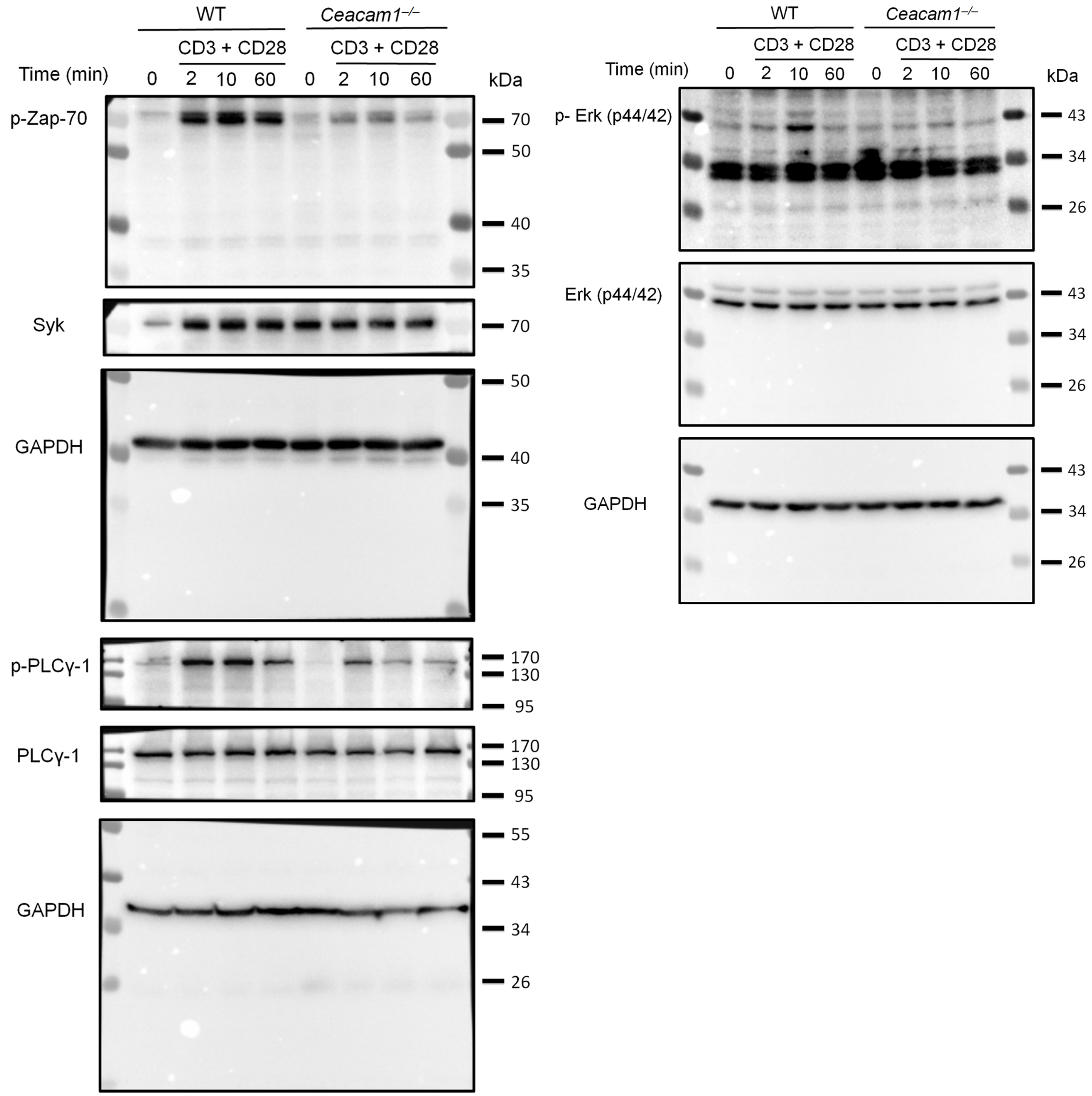

Supplementary Figure 5. Uncropped Western blots shown in Figure 3. Protein samples were loaded on 10% SDS-PAGE gels and transferred onto nitrocellulose membrane by standard techniques. The membranes were then developed from top to bottom, respectively.

Supplementary Fig. 6

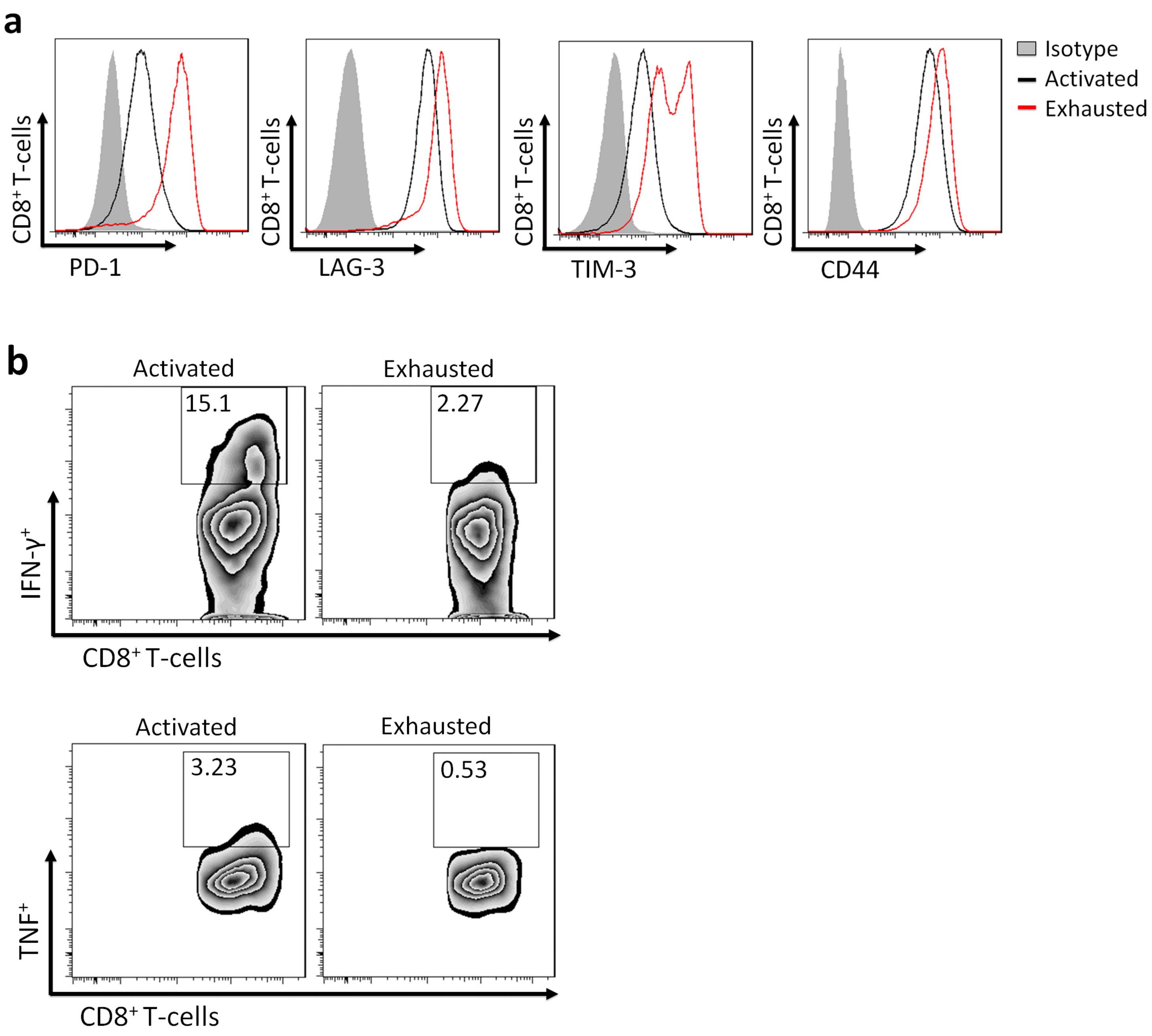

Supplementary Figure 6. *In vitro* exhausted T cells express high levels of inhibitory receptors and lose their function of cytokine production.

a: Representative FACS histograms showing expression levels of PD-1, LAG-3, TIM-3, and CD44 in cultured $CD8^+$ T cells from naïve P14 × wild-type (WT) mice given an activating (black line) or an exhausting (red line) dose of GP33 peptide, as measured by flow cytometry. Staining with isotype control antibody is shown as grey area (n = 6 per group). **b:** Representative FACS histograms showing secretion of intracellular cytokine interferon (IFN)-γ and tumor necrosis factor alfa (TNF) of cultured $CD8^+$ T cells from naïve P14 × WT mice given an activating or an exhausting dose of GP33 peptide, as measured by flow cytometry (n = 4-6 per group). Data are representative of three (**a** & **b**) experiments.

Supplementary Fig. 7

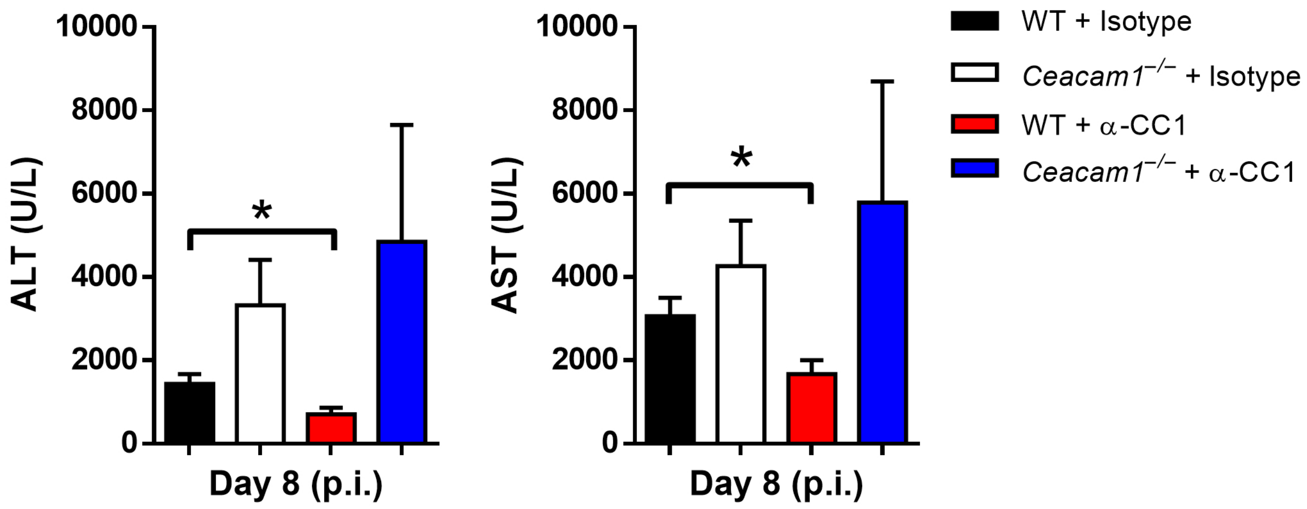

Supplementary Figure 7. Treatment with anti-CEACAM1 mAb reduces immunopathology. Groups of wild-type (WT) and *Ceacam1*$^{-/-}$ mice were given equal amounts of either anti-CEACAM1 monoclonal antibody (mAb; clone CC1) or isotype antibody on day -1 (100 µg per mouse) and on day 3 (200 µg per mouse). All mice were infected with 2×10^4 PFU of LCMV-Docile on day 0. Graphs show the activity of serum alanine transaminase (ALT) and aspartate aminotransferase (AST) in indicated groups on day 8 after infection (n = 3 per group; mean ± SEM).
